# A Complication following the Transcatheter Closure of a Muscular Ventricular Septal Defect

**Published:** 2015-07-03

**Authors:** Mustafa Karaçelik, Pelin Öztürk, Onur Doyurgan, Uğur Karagöz, Murat Muhtar Yilmazer, Timur Meşe, Osman Nejat Sariosmanoğlu

**Affiliations:** 1 *Department of Pediatric Cardiac Surgery, Dr. Behçet Uz Children’s Hospital, Izmir, Turkey.*; 2 *Department of Pediatric Cardiology, Dr. Behçet Uz Children’s Hospital, Izmir, Turkey.*

**Keywords:** *Heart septal defect*, *ventricular*, *Septal occluder device*, *Endocarditis*

## Abstract

Today, congenital heart diseases may be treated without surgery through advances in interventional cardiology. However, complications such as infection and thrombus formation may develop due to foreign materials used during these procedures. Surgical intervention may be required for the removal of the device utilized for the procedure.

In this case report, we present the surgical treatment of a residual ventricular septal defect (VSD) that had developed in a 6-year-old patient with an apical muscular VSD closed with the Amplatzer muscular VSD device. The patient was admitted to the emergency room with complaints of abdominal pain and high fever 5 days after discharge without any cardiac symptoms. When she arrived at our clinic, she had a heart rate of 95 bpm, blood pressure of 110/70 mmHg, and temperature of 38.5ºC. Examinations of the other systems were normal, except for a 3/6 pan-systolic murmur at the mesocardiac focus on cardiac auscultation. Echocardiography showed a residual VSD, and the total pulmonary blood flow to the total systemic blood flow ratio (Qp/Qs) of the residual VSD was 1.8. In the operating room, the Amplatzer device was removed easily with a blunt dissection. The VSD was closed with an autologous fresh pericardial patch. Following the pulmonary artery debanding procedure, the postoperative period was uneventful. The condition of the patient at the time of discharge and in the first postoperative month’s follow-up was good. There was no residual VSD or infection.

## Introduction

Some congenital heart anomalies no longer require surgical treatment and are treated via percutaneous interventions thanks to advances in interventional cardiology. Nowadays, perimembranous and muscular ventricular septal defects (VSDs) can be closed by the same methods following the transcatheter closure of atrial septal defects and patent foramen ovales in the past.^1^ However, as is the case in every interventional procedure, complications such as infection and thrombus formation may develop when a foreign device is inserted into the heart.^2^

In this case report, we present the surgical treatment of a residual VSD in a 6-year-old patient with an apical muscular VSD closed with the Amplatzer muscular VSD device.

## Case Report

The diameter of the apical muscular VSD was measured as 8.5 mm on transthoracic echocardiography performed for a one-year-old girl who was born through a normal pregnancy process and delivery, weighing 3000 grams. The total pulmonary blood flow to total systemic blood flow ratio (Qp/Qs) was 2.2 on echocardiography. She had undergone pulmonary artery banding operation via left thoracotomy in another center in 2009. The patient was admitted to our hospital for a complete correction operation when she was 6 years old, and a decision was made to close the VSD with the Amplatzer device and surgical pulmonary artery debanding thereafter in the cardiology-pediatric cardiac surgery council. The apical muscular VSD was closed with a 10-mm Amplatzer muscular VSD occluder device (AGA Medical Corporation, Golden Valley, MN, USA) under general anesthesia in the angiography laboratory of interventional cardiology after an informed consent had been obtained from the family. Intravenous Cefazolin sodium (100 mg/kg single dose) was administered 30 minutes before the procedure. The procedure was completed unproblematically. A moderate residual shunt across the VSD device was observed on transthoracic echocardiography performed after the procedure. The patient was discharged from the hospital with recovery after arrangement of the antibiotherapy one day after the procedure. Acetylsalicylic acid (Aspirin®, Bayer HealthCare LLC, Germany) tablet at a dose of 2 mg/kg/day and s*ubacute bacterial endocarditis* prophylaxis for 6 months were advised. Debanding was planned to be performed one month later. 

However, the patient was admitted to the emergency room with complaints of abdominal pain and high fever 5 days after discharge. On her physical examination, she had a heart rate of 95 beats per minute (bpm), blood pressure of 110/70 mmHg, and temperature of 38.5 ºC. Other system examinations were normal, except for a 3/6 pan-systolic murmur at the mesocardiac focus on cardiac auscultation. Laboratory test results were as follows: white blood cell (WBC) count of 19230/mm^3^ with 79.9% polymorphonuclears (reference = 43-65%) and 12.2% lymphocytes (reference = 20.5- 45.5%); hemoglobin of 11.5 g/dL; platelet count of 276000/mm^3^; C-reactive protein (CRP) of 19.83 mg/dL (previous value = 1.18 mg/dL, reference = 0-0.5 mg/dL); erythrocyte sedimentation rate (ESR) of 119 mm/hour (previous value = 7 mm/hour); proBNP (brain natriuretic peptide) of 2040 pg/ml (reference < 300 pg/ml); and creatine phosphokinase MB isoenzyme of 28 IU/L (reference = 0-24 IU/L). A total of five blood cultures were negative. Additionally, pharynx and urinary cultures were obtained, and no growth was determined. Urine analysis showed no pathology. Sinus tachycardia was detected on the electrocardiogram (110 bpm). No vegetation was detected around the VSD on transthoracic echocardiography. A residual VSD and an increase in the left-to-right shunting were detected. The Qp/Qs ratio of the residual VSD was 1.8. Despite the negative blood cultures, device dehiscence developing secondarily to endocarditis was considered. Intravenous Piperacillin/Tazobactam (200 mg/kg qid), Gentamicin (5 mg/kg bid), and Vancomycin (50 mg/kg tid) treatment was started on the same day. 

**Figure 1:**
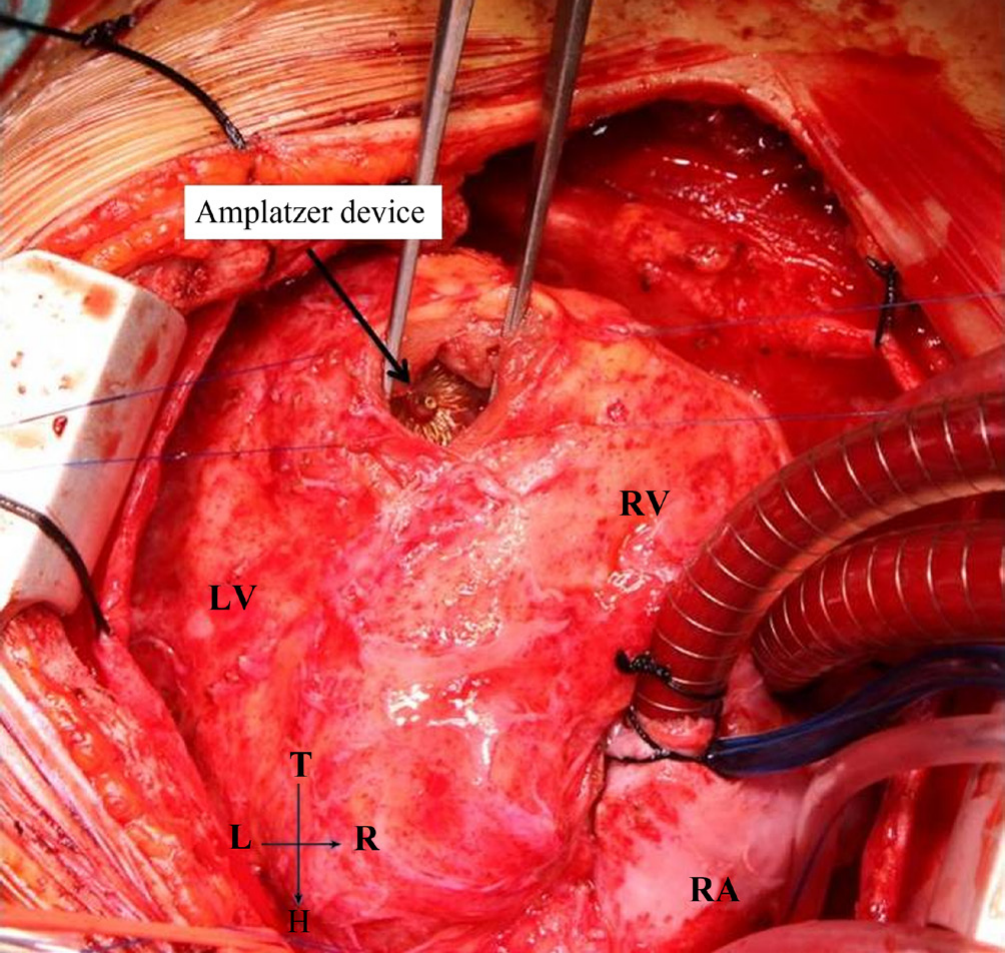
Intraoperative appearance of the apical muscular ventricular septal defect and Amplatzer device (AGA Medical Corporation, Golden Valley, MN, USA)

RV, Right ventricle; LV, Left ventricle; RA, Right atrium; R, Right; L, Left; H, Head; T, Toe

An urgent operation was decided for the removal of the Amplatzer device, closure of the VSD, and debanding of the pulmonary artery due to the presence of remittent fever and CRP of 18.86 mg/dL on day 9 of antibiotic therapy. The patient underwent an operation 21 days after the installation of the Amplatzer device after informed consent had been obtained. Cardiopulmonary bypass was performed following the standard aorta bicaval cannulation under general anesthesia. Hypothermia was applied at 32 ºC. Cold blood cardioplegia was provided twice (+4 ºC) following aortic cross-clamping, and a right ventriculotomy was performed with a longitudinal incision close to the apex of the right ventricle and in parallel with the septum. The Amplatzer device was accessed, and it was observed that it had closed the VSD completely. However, the device was completely dehiscenced from the rim of the VSD in half of the inferior part of the septum. Apart from this dehiscence, a mild tissue injury was observed, and the remaining half part was seen to be unproblematic and integrated with the rim of the VSD and epithelized (Figure 1). The Amplatzer device was removed easily with a blunt dissection; the septum and neighboring tissues were irrigated after culture had been obtained from the tissue specimens. First, the VSD was closed with an autologous fresh pericardial patch (2 × 1.5 cm) and individual 5.0 pledget Prolene stitches. Afterwards, two felts (3 × 0.5 cm) were formed with the autologous fresh pericardial patch for support to use when closing the ventriculotomy; the right ventriculotomy was closed with 5.0 Prolene sutures with the over-and-over technique. The narrowing part of the pulmonary artery was excised following the pulmonary artery debanding procedure and an end-to-end anastomosis was closed with 5.0 Prolene sutures. The operation was completed with no complications. The patient was extubated on the postoperative day one and transferred to the ward. She did not have a fever on her postoperative follow-up. She had CRP of 1.18 mg/dL, ESR of 12 mm/hour, and WBC count of 6700/mm^3^. 

The patient's postoperative course was unproblematic, and no growth occurred in the tissue cultures obtained intraoperatively. In addition, no residue was detected in the VSD. She was discharged after recovery following 3 weeks of intravenous Piperacillin/Tazobactam, Gentamicin, and Vancomycin treatment. The patient had no problems in the first postoperative month’s follow-up.

## Discussion

In recent years, transcatheter VSD closure in experienced clinics has been accepted as an alternative to surgical treatment due to its availability and lower complication rates, particularly in muscular VSDs. The most common complication is arrhythmia in VSDs in which the transcatheter device is inserted. Rarely, embolism and endocarditis may also be observed.^3^ The Amplatzer device-related endocarditis is a problem, which should be immediately treated as it is a life-threatening complication.^2^^, ^^4^

The foreign body should be removed from the tissue and also an autologous fresh pericardial patch should be used instead of the removed foreign body in cases which do not respond to antibiotic treatment. While right atriotomy is preferred for accessing the VSD in the surgical closure of perimembranous VSDs, right or left ventriculotomy is preferred in apical muscular VSDs.^5^

When an operation is planned for a residual VSD in patients whose apical muscular VSD is closed with the device, we would suggest right ventriculotomy through a longitudinal incision in parallel with the septum, as was the case in our patient, because it facilitates explorations and shortens surgery time.

## Conclusion

As a conclusion, when there is a possibility of endocarditis following an unsuccessful transcatheter closure of an apical muscular VSD, it is vital that the patient undergo urgent surgery for device removal. 

## Notes:

***This paper should be cited as:*** Karaçelik M, Öztürk P, Doyurgan O, Karagöz U, Yilmazer MM, Meşe T, Sariosmanoğlu ON. A Complication following the Transcatheter Closure of a Muscular Ventricular Septal Defect. J Teh Univ Heart Ctr 2015;10(3):149-151.
